# The Role of NEU1 in Coronavirus Infection and Pathogenesis

**DOI:** 10.23880/vij-16000351

**Published:** 2024-08-08

**Authors:** Y Wu, GY Chen

**Affiliations:** Children’s Foundation Research Institute at Le Bonheur Children’s Hospital, Department of Pediatrics, University of Tennessee Health Science Center, USA

**Keywords:** SARS-CoV-2, COVID-19, Sialylation, Sialidases

## Abstract

Severe acute respiratory syndrome coronavirus 2 (SARS-CoV-2) has caused the coronavirus disease 2019 (COVID-19) pandemic, resulting in millions of infections and deaths worldwide. Although vaccines are available, they appear to be less efficacious against newly emerging variants of the virus. Thus, therapeutic modalities are urgently needed. The coronavirus genome encodes four major structural proteins: the spike (S) protein, nucleocapsid (N) protein, membrane (M) protein, and envelope (E) protein, all of which are required to produce a structurally complete viral particle. N protein is one of the most abundant structural proteins, participates in the regulation of viral replication and virion assembly, and is a major immunogen in coronavirus infection-induced disease. Sialylation is the addition of sialic acids to the terminal glycans of glycoproteins and glycolipids, which act as key components for biological functions of glycoproteins or glycolipids. Sialidases (or neuraminidases) are glycosidases that remove sialic acid residues (desialylation) from glycan portions of glycoproteins or glycolipids. Through desialylation, sialidases modulate the functionality of sialic acid-containing molecules and are involved in both physiological and pathological pathways. This review aims to explore the current understanding of NEU1’s involvement in coronavirus infection and pathogenesis, synthesizing available research and identifying areas for future investigation.

## Introduction

Coronaviruses have emerged as significant threats to global public health, as evidenced by the recent COVID-19 pandemic caused by SARS-CoV-2. These viruses are known for their ability to cause respiratory infections in humans and animals, ranging from mild common colds to severe acute respiratory syndromes [1]. As research into coronavirus pathogenesis intensifies, attention has turned to host factors that may influence viral entry, replication, and disease progression [2].

Neuraminidase 1 (NEU1), a lysosomal sialidase enzyme that plays a crucial role in the catabolism of sialoglycoconjugates. NEU1 has been implicated in various physiological and pathological processes, including inflammation, immune responses, and cellular signaling [3]. Recent studies have suggested that NEU1 may also play a significant role in coronavirus infections, making it an important subject of investigation in the field of virology and potential therapeutic interventions [2].

### A Remaining, Unmet need for Effective Therapeutics against COVID-19

The COVID-19 pandemic, caused by severe acute respiratory syndrome coronavirus 2 (SARS-CoV-2), has claimed millions of lives worldwide. Whereas the WHO has declared COVID-19 is no longer a global health emergency, the virus continues to infect and spread, ravaging communities and imposing a significant burden on health care systems. Although vaccines are available, they appear to be less efficacious against newly emerging variants of the virus [4–9]. Furthermore, SARS-CoV-2 is likely to become endemic, leading to the emergence of vaccine-escape variants and reinforcing the need for development of antiviral therapeutics. SARS-CoV-2 is a β-coronavirus with a large (30 Kb) positive-strand RNA genome [10]. Although several antivirals are available for treating COVID-19, there are limitations for each treatment. Remdesivir, approved by the FDA for treatment of hospitalized COVID-19 patients over the age of 12 years, only modestly accelerates recovery [11]. Many of the monoclonal antibody regimens that were effective against past variants are of limited efficacy against the SARS-CoV-2 Omicron variant [12–17]. More recently, Pfizer’s co-administered antiviral treatment of Paxlovid (nirmatrelvir and ritonavir) was approved by the FDA for use in children age 12 and above, but its effectiveness against SARS-CoV-2 variants is unclear. Thus, the development of novel therapeutics against SARS-CoV-2 remains a top priority for combating the current pandemic and future coronavirus outbreaks.

The coronavirus genome encodes four major structural proteins: spike (S), nucleocapsid (N), membrane (M), and envelope (E). All four proteins are required to produce a structurally complete viral particle [18,19]. SARS-CoV-2 pathogenesis is expanding rapidly, most studies have focused on a few proteins, such as the S protein, and on how it interacts with the host immune systems. Less is known about the mechanisms that globally control host and viral replication during infection. As one of the most abundant structural proteins, the N protein plays key roles in regulating viral replication and virion assembly and is a major immunogen in coronavirus-induced disease. Therefore, N is an attractive viral target for diagnosis and treatment strategies against HCoVs including SARS-CoV-2 [19,20]. Nevertheless, the mechanisms by which specific cellular genes are induced and regulated during SARS-CoV-2 infection are poorly understood. Knowledge of how host defense genes are controlled might be key to understanding COVID-19 pathogenesis.

### Exacerbated Inflammatory Response in SARS-CoV-2 Infection

While the pathophysiology of SARS-CoV-2 remains to be fully characterized, the infection triggers an exacerbated inflammatory response, as indicated by elevated levels of various proinflammatory cytokines (IL-6, −1β, −12, −18, IFNα, IFN-γ, TNFα, and TGFβ) [21–23]. Clinically, this escalated inflammatory response can lead to cytokine storm and sepsis, which cause multiorgan failure and contribute to 28% of deaths due to COVID-19 [24]. Therefore, treatments that can suppress host inflammatory responses might provide effective therapeutic strategies for COVID-19.

### Sialylation and Desialylation Play Important Roles in Various Biological Processes

**Figure 1:**
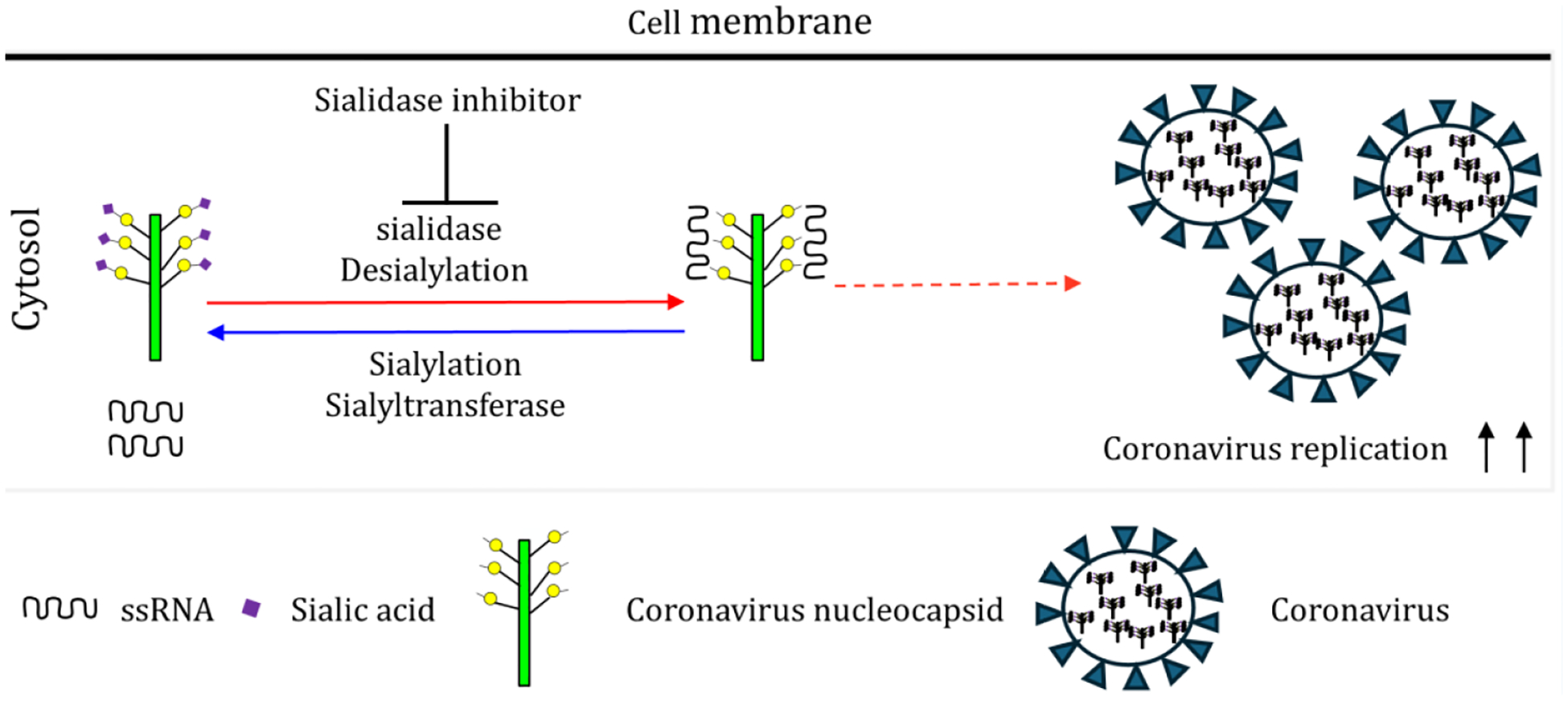
Sialylation of coronavirus nucleocapsid (N) protein is critical for its RNA binding activity and viral replication.

Sialylation, the most frequent modification of proteins and lipids, is the addition of sialic acids (a family of nine-carbon acidic monosaccharides) to the terminal residues of the glycan moieties of glycoproteins and glycolipids as shown in Figure 1. This modification is important in self-nonself discrimination, phagocytosis of cancer cells by macrophages, determination of immunoglobulin E allergic pathogenicity, and bacterial infection [25–29]. The sialylation level of a cell is largely dependent on the activity of two kinds of enzymes: sialyltransferases that add sialic acid residues to glycolipids or glycoproteins and sialidases that remove sialic acid residues from glycolipids or glycoproteins as shown in Figure 1 [28]. Desialylation as a means of modulating the functionality of sialic acid-containing molecules is often involved in signal transduction in either physiological or pathological processes.

### Sialidase Inhibitors in Infection Therapeutics

Sialidase inhibitors are useful tools for studying sialidase function and serve as drugs for sialidase-related diseases, such as viral infection. Inhibition of viral neuraminidase activity has been successfully utilized as a therapeutic approach for influenza infection [30]. Tamiflu (oseltamivir) and Relenza (Zanamivir), which are approved for treatment of influenza A and B, have almost no effect on human sialidases (neuraminidases) but are potent inhibitors of neuraminidase (NA) activity of the influenza NA protein [30,31]. Several clinical trials assessed the efficacy of oseltamivir in treating SARS-CoV-2 infection but no positive outcomes were observed [32–36]. This lack of efficacy could be attributed to several reasons: 1) SARS-CoV-2 genomic RNA does not encode a sialidase activity; 2) SARS-CoV-2 replication depends on the host cellular sialidase Neu1; and 3) oseltamivir has few inhibitory effects on host Neu1 [31,37,38]. Moreover, more recently studies indicated that sialidase inhibitors rescued mice from bacterial infection-induced death by inhibiting the activity of host cell surface sialidase Neu1 and suppressing the cytokine storm [39].

### Sialylation of Coronavirus Nucleocapsid (N) Protein is Critical for its RNA Binding Activity and Viral Replication

N proteins from SARS-CoV-2 and human coronavirus HCoV-OC43 were significantly sialylated and this sialylation was tightly regulated by host Neu1 [2]. Coronavirus replication occurs in the cytoplasm of infected host cells [40]. The sialidase inhibitor Neu5Ac2en-OAcOMe that targets cytoplasmic but not cell surface sialidases, which is advantageous because β-coronaviruses traffic to lysosomes where Neu1 is predominantly localized [3,41–43]. Notably, the sialidase inhibitor Neu5Ac2en-OAcOMe reduced replication of HCoV-OC43 and SARS-CoV-2 *in vitro* and *in vivo* by inhibiting host Neu1 activity. Therefore, sialidase inhibitors could be a generalizable and effective treatment in the current COVID-19 pandemic and future pandemics associated with the inflammatory response as shown in Figure 1.

## Conclusion

Neu1 regulates coronavirus replication by controlling sialylation on coronavirus nucleocapsid protein. Coronavirus nucleocapsid proteins in COVID-19 patients and in coronavirus HCoV-OC43-infected cells were heavily sialylated; this sialylation controlled the RNA binding activity and replication of coronavirus. While many aspects of NEU1’s involvement remain to be fully elucidated, its position at the intersection of viral-host interactions and immune responses makes it a promising subject for further research.

Understanding the role of NEU1 in coronavirus infections could not only enhance our knowledge of viral pathogenesis but also open new avenues for therapeutic interventions. As the world continues to grapple with the challenges posed by coronaviruses, exploring host factors like NEU1 may provide crucial insights for developing more effective strategies to combat these formidable pathogens.
